# Synthesis and Characterization of Silver-Doped Mesoporous Bioactive Glass and Its Applications in Conjunction with Electrospinning

**DOI:** 10.3390/ma11050692

**Published:** 2018-04-28

**Authors:** Francesca E. Ciraldo, Liliana Liverani, Lukas Gritsch, Wolfgang H. Goldmann, Aldo R. Boccaccini

**Affiliations:** 1 Institute of Biomaterials, Department of Materials Science and Engineering, University of Erlangen-Nuremberg, Cauerstraße 6, 91058 Erlangen, Germany; francesca.elisa.ciraldo@fau.de (F.E.C.); liliana.liverani@fau.de (L.L.); lukas.gritsch@fau.de (L.G.); 2 Institute of Biophysics, Department of Physics, University of Erlangen-Nuremberg, Henkestraße 91, 91052 Erlangen, Germany; wgoldmannh@aol.com

**Keywords:** bioactive glass, ordered mesoporous glass, silver, antimicrobial, electrospinning, scaffolds

## Abstract

Since they were first developed in 2004, mesoporous bioactive glasses (MBGs) rapidly captured the interest of the scientific community thanks to their numerous beneficial properties. MBGs are synthesised by a combination of the sol–gel method with the chemistry of surfactants to obtain highly mesoporous (pore size from 5 to 20 nm) materials that, owing to their high surface area and ordered structure, are optimal candidates for controlled drug-delivery systems. In this work, we synthesised and characterised a silver-containing mesoporous bioactive glass (Ag-MBG). It was found that Ag-MBG is a suitable candidate for controlled drug delivery, showing a perfectly ordered mesoporous structure ideal for the loading of drugs together with optimal bioactivity, sustained release of silver from the matrix, and fast and strong bacterial inhibition against both Gram-positive and Gram-negative bacteria. Silver-doped mesoporous glass particles were used in three electrospinning-based techniques to produce PCL/Ag-MBG composite fibres, to coat bioactive glass scaffolds (via electrospraying), and for direct sol electrospinning. The results obtained in this study highlight the versatility and efficacy of Ag-substituted mesoporous bioactive glass and encourage further studies to characterize the biological response to Ag-MBG-based antibacterial controlled-delivery systems for tissue-engineering applications.

## 1. Introduction

Bioactive glasses (BGs) are noncrystalline materials that are able to bond with living tissue and stimulate new tissue growth [1,2]. Because of their high biomineralization ability, biodegradability, osteogenic, and angiogenic potential, BGs are promising candidates for tissue-engineering applications [2]. They have the capability to bond to both soft and hard tissues and to degrade once in contact with biological fluids. The most widely studied BG composition is bioactive silicate glass with a composition (in wt. %) of 45% SiO_2_, 24.5% CaO, 24.5% Na_2_O, and 6% P_2_O_5_ [3,4].

Two processing methods are traditionally applied to synthesize BGs: the melt-quenching process and the sol–gel technique. Moreover, in 2004 a new family of sol–gel glasses was developed by Yan et al. [5] through the combination of the supramolecular chemistry of surfactants and the sol–gel method. These glasses, called ordered mesoporous bioactive glasses (MBGs), are usually based on the SiO_2_–CaO–P_2_O_5_ system. They are characterised by faster mineralization compared to traditional sol–gel and melt-derived ones, large specific surface area, tunable and ordered mesoporosity, and a pore size ranging between 5 and 20 nm [6,7,8]. These unique features make MBGs optimal candidates to be used as local drug-delivery systems for drugs, biomolecules, or therapeutic metallic ions [6,7,9]. In the last decades, several metallic ions such as copper, gallium, strontium, silver, cobalt, cerium, and zinc have been incorporated in bioactive glasses to enhance bone formation due to their effect on osteogenesis and angiogenesis [10,11,12].

Notably, silver and gallium have attracted particular interest, as they have been reported to give a strong antibacterial effect to MBGs [13,14,15,16]. Ag-doped sol–gel BGs have been studied for over 10 years [13,14,15]. Silver is well known for its antimicrobial properties, causing the detachment of the cytoplasm membrane of bacteria from the cell wall, compromising the bacteria’s ability to replicate [14].

MBGs can also be used to fabricate three-dimensional porous scaffolds. The first highly porous bioactive glass–ceramic 3D scaffolds based on melt-derived 45S5 bioactive glass were developed in 2006 by Chen et al. [17]. They fabricated scaffolds with porosity >90% using a standard foam replica technique and polyurethane foams as sacrificial template. In 2007, the replication technique was applied in combination with MBGs, leading to scaffolds with an interconnected macroporous network and uniform mesoporous struts [18]. A few preliminary studies have also demonstrated that the application of a MBG coating can reinforce the mechanical properties of pristine scaffolds [19]. MBGs have been also applied as a coating on different kinds of foam-like structures, obtaining multifunctional systems with high bioactivity, improved mechanical properties (e.g., compressive strength similar to those of trabecular bone; 2–12 MPa), and drug-release capability [19,20,21].

In the present work, the synthesis and characterization of a novel silver-containing MBG (Ag-MBG) is reported. The efficacy of the ion doping and the bioactive and antibacterial properties of the MBG against both Gram-positive *Staphylococcus Carnosus* and Gram-negative *Escherichia coli* were investigated. Moreover, a feasibility study of possible applications of the synthesised Ag-MBG in conjunction with electrospinning or electrospraying was carried out. These include: (i) fabrication of submicron electrospun composite-fibre mats by dispersing Ag-MBG particles in a poly(ε-caprolactone) (PCL) solution; (ii) coating of Ag-MBG on bioactive glass-based scaffolds by electrospraying; and (iii) direct electrospinning of the Ag-containing silicate sol. The results confirm the versatility and efficacy of Ag-substituted mesoporous bioactive glasses and support the development of antibacterial technologies for tissue-engineering scaffolds based on Ag-MBG.

## 2. Materials and Methods

### 2.1. Synthesis of Ag-MBG

Ion-doped mesoporous bioactive glass with a composition (in mol %:) 78% SiO_2_, 20% CaO, 1.2% P_2_O_5_, and 0.8% Ag_2_O was synthesised according to the protocol reported by Philippart et al. [10]. The nonionic surfactant Pluronic^®^ F127 was used as a structure directing agent and the evaporation-induced self-assembly (EISA) process was employed for the production of the mesoporous silver-doped glass (Ag-MBG). Briefly, 4.5 g of Pluronic F127 (Sigma-Aldrich, Steinheim, Germany) was dissolved overnight in 85 mL of ethanol (VWR Chemicals, Darmstadt, Germany) and 1.2 mL of HNO_3_ 1M (Sigma-Aldrich, Steinheim, Germany). Glass precursors were added with a 3 h interval between each addition, under continuous stirring, in the following order: tetraethyl orthosilicate (TEOS), triethyl phosphate (TEP), calcium nitrate (Ca(NO_3_)2·4H_2_O), and silver nitrate. All reagents were purchased from Sigma-Aldrich, Germany and used without further purification. The solution was stirred for 24 h at room temperature (RT) and then poured into petri dishes (20 mL/petri dish) for EISA for 5–7 days. The obtained dried gels were then calcined at 700 °C for 3 h. The resulting samples were ground to obtain fine powders of 20 µm average particle size.

### 2.2. Bioactivity Study in SBF

To assess the ability of the MBG to form a layer of hydroxycarbonate apatite (HCA), the mineral phase of natural bone, upon immersion in simulated body fluid (SBF), which is the marker of bioactivity, Ag-MBG powder was added to SBF, which was prepared following the procedure reported by Kokubo and Takadama [22]. For SBF preparation, 8.035 g L^−1^ NaCl, 0.355 g L^−1^ NaHCO_3_, 0.255 g L^−1^ KCl, 0.231 g L^−1^ K_2_HPO_4_ (3H_2_O), 0.311 g L^−1^ MgCl2 (6H_2_O), 0.292 g L^−1^ CaCl_2_, and 0.072 g L^−1^ Na_2_SO_4_ were dissolved in deionised water and buffered at pH 7.4 at 36.5 °C using 6.118 g L^−1^ tris(hydroxymethyl) aminomethane ((CH_2_OH)_3_CNH_2_)) and 1 M HCl. Glass powders were soaked in SBF with a concentration of 1.5 g L^−1^ according to the protocol reported by Maçon et al. [23]. SBF immersion tests allow for studying the ability of bioactive glasses to form a layer of HCA on their surface in a physiological-like environment. The samples were placed in an orbital shaker (IKA^®^ KS 4000i control, Staufen, Germany) at 37 °C for up to 7 days. The solution was renewed every 2 days in order to better simulate in vivo conditions [24]. At each time point, powders were rinsed with deionised water and centrifuged (Eppendorf 5430 microcentrifuge, Eppendorf, Germany) in order to separate the particles from the liquid and dried overnight at 60 °C.

### 2.3. Characterisation of Ag-MBG

Scanning electron microscopy (SEM) micrographs of the sample were recorded with an Auriga SEM instrument (Zeiss, Oberkochen, Germany). Energy dispersive spectroscopy (EDS) (Oxford Instruments, Abingdon, UK) was carried out in order to qualitatively assess the presence of silver in the glass. For transmission electron microscopy (TEM) analysis, the samples were placed on a lacey carbon grid. To study the inner microstructure of the material, a JEOL-3010 transmission electron microscope operating at an acceleration voltage of 300 kV was used. Images were taken using a CCD camera (model Keen view, SIS analysis size 1024 × 1024, pixel size 23.5 mm × 23.5 mm).

Nitrogen physisorption isotherms were measured at 77 K using a Micrometrics ASAP 2010 porosimetry instrument. In order to perform the measurement, the sample was outgassed at 120–150 °C for 12 h under vacuum. The specific surface area was calculated from the BET equation in the linear range between 0.07 and 0.23 P/P0.

X-ray fluorescence (XRF, Zetius, PANalytical) was used to verify the silver incorporation and to characterize the chemical composition of the glass.

After the immersion period in SBF, the samples were analysed by means of an array of techniques: (i) Fourier transform infrared spectroscopy (FTIR) (4000–400 cm^−1^, 32 scans, Nicolet 6700, Thermo Scientific, Waltham, MA, USA); (ii) X-ray diffraction (XRD) (X’Pert Pro diffractometer with Cu-Kαradiation at 40 mV and 40 mA, 2θ = 10–80°, step size 0.03 s^−1^, Philips); (iii) SEM; and (iv) Raman spectroscopy (He-Ne laser at 632 nm, LabRAM HR800, Horiba Jobin Yvon, Kyoto, Japan). Samples as produced were used as reference. SBF was kept for the analysis of the concentration of Ca, P, Si, and Ag ions by the use of inductively coupled plasma optical emission spectrometry (ICP-OES, Varian 710-ES).

Preliminary antibacterial agar diffusion tests were carried out against *Staphylococcus Carnosus* (Gram-positive) and *Escherichia Coli* (Gram-negative) bacteria. A bacteria population was prepared, suspending it in a lysogeny broth (LB) medium, and its optical density (OD) was adjusted (600 nm, Biophotometer Plus, Eppendorf AG, Hamburg, Germany) to reach the value of 0.015, according to a previously developed protocol. Then, 20 µL of the prepared medium was deposited and spread homogeneously onto a petri dish of 10 cm diameter, which was previously covered with a uniform layer of LB Agar. The samples (Ag-MBG pellets) were then settled on the agar and the culture was incubated for 24 h at 37 °C and high relative humidity (~80%). After the incubation time, the possible inhibition zones around the samples were assessed.

### 2.4. Electrospinning Process

Fibrous scaffolds were obtained using a commercial electrospinning device (EC-CLI, IME Technologies Netherlands), with accurate control of temperature and relative humidity (RH). For all the samples, the set values were 23 °C and 40% RH. The optimization of the Taylor cone was obtained using a gas shield accessory with nitrogen flux set at 8 mL/min. For the coating of scaffolds with electrospun fibres, the starting solution was a mixture in ratio 1:1 of the sol–gel solution containing glass precursors (as described in paragraph 2.1) and a 10% *w*/*v* solution of poly vinyl alcohol (PVA) (72 kDa, AppliChem, Panreac, Germany) in deionised water. The mixture was fed at 0.5 mL/h by using a needle of 21G. The applied voltage was set at 20 kV and the distance between the needle and the scaffold (used as target) was 10 cm.

To develop Ag-MBG–PCL composite fibres, Ag-MBG particles were sieved to a size lower than 20 μm before the addition to the PCL (80 kDa, Sigma Aldrich, Germany) solution. Ag-MBG particles were dispersed in a solution of PCL dissolved at the concentration of 20% *w*/*v* in acetic acid (VWR, Darmstadt, Germany). The amount of Ag-MBG particles, 30% *w*/*w* with respect to the polymer amount, was selected according to the literature [25] to preserve bioactive glass bioactivity after the incorporation in the polymer matrix. However, since this solution did not have rheological properties suitable for electrospinning, the amount of added Ag-MBG particles was reduced to 5% *w*/*w*. The solution was then fed at 0.8 mL/h through a 23G needle. The distance between the needle and the target was fixed at 15 cm and the applied voltage was +13 kV at the needle and −2.5 kV at the target. Average fibre diameter (calculated on 50 fibres) and pore size were evaluated using the software Fiji and its plugin DiameterJ [26].

### 2.5. Fabrication of 45S5 BG Based Scaffolds

The starting material was melt-derived 45S5 bioactive glass (BG, nominal particle size 2 μm, Schott Vityxx^®^, Mainz, Germany). Marine sponges *Spongia Agaricina* harvested in the Indo-Pacific Ocean (Pure Sponges, Solihull, UK) were selected as a sacrificial template [27]. Bioactive glass-based scaffolds were fabricated by the foam replica technique, introduced by Chen et al. [17]. Briefly, polyvinyl alcohol (PVA) was dissolved in deionised water at 80 °C for 1 h. After complete dissolution of the polymer, bioactive glass (40 wt. %) was added to the polymeric solution and it was let to disperse homogeneously. Several sponge specimens were cut in a standard cylindrical shape of Ø = 8 mm and h = 8 mm and soaked in the PVA/BG slurry for 10 minutes. Subsequently, the excess of slurry was removed and the coated foams were allowed to slowly air-dry at room temperature. A second BG layer was then applied using the same immersion procedure. This time, the excess of slurry was removed with compressed air, as previously reported [27]. Once the samples were dried, they underwent a heat treatment in order to burn out the sacrificial template and to sinter the BG struts. The sintering conditions were 400 and 1050 °C for 1 h, respectively, with a heating rate of 2 °C/min.

The resulting scaffolds were then coated directly by electrospinning using the Ag-MBG sol as spinning solution. The selected parameters were a flow rate of 2 mL/h, an applied voltage of 20 kV, and a spinning time of 1 h. The scaffolds were dried at 60 °C overnight and calcined at 700 °C for 3 h.

## 3. Results

### 3.1. Microstructural and Chemical Characterisation of Ag-MBG

**Figure 1:**
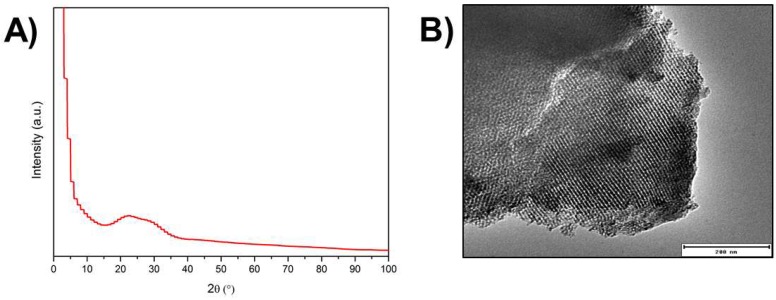
XRD pattern of the Ag-MBG developed in this study (**A**) and TEM micrograph showing the ordered mesoporosity of Ag-MBG (**B**).

From the analysis of the XRD pattern performed on synthesised silver-doped mesoporous glasses (Figure 1A), it was possible to verify the absence of diffraction peaks, indicating that the sample is amorphous. A broad peak in the range 2θ = 20°–35° is visible, denoting that no crystalline phase has developed during the heat treatment.

**Figure 2:**
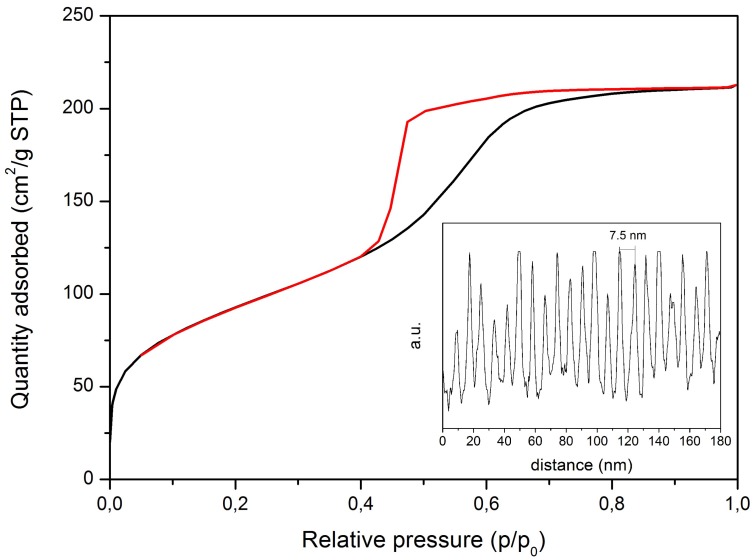
Nitrogen adsorption isotherm of Ag-MBG and pore size calculated by ImageJ (inset).

Figure 1B shows a typical TEM micrograph of the synthesised Ag-MBG. After investigation of different particles, it was possible to conclude that Ag-MBG is characterised by an ordered 2-D hexagonal mesoporous structure. Nitrogen physical analysis was carried out to determine the specific surface area and pore volume of Ag-MBG. Pore size was qualitatively calculated using the software ImageJ freeware (National Institutes of Health, Bethesda, MD, USA). Results are shown in Figure 2. The curve can be identified as type IV isotherm, which is characteristic of mesoporous materials [6,10,28].

The hysteresis loop obtained is a type H2, which is characteristic of ordered porous materials [6,9,29]. The BET surface area reached 345 m^2^/g and the total pore volume calculated at P/P0 = 0.99 was found to be 0.423 cm^3^/g. The pore size was calculated to be around 7.5 nm. These results are in agreement with the literature [6], where it is reported that mesoporous glasses are characterised by a SBET in a range between 200 and 500 m^2^/g and a pore size in a range of 5–20 nm. The presence of silver was detected in the samples by XRF analysis (Table 1), confirming the incorporation and maintenance of this element in the vitreous matrix after thermal treatment at 700 °C. The presence of other trace elements (e.g., SrO, CuO, Cl) (ppm) was detected, which is probably due to contamination that occurred during the glass synthesis.

### 3.2. Acellular Bioactivity Test in SBF

**Figure 3:**
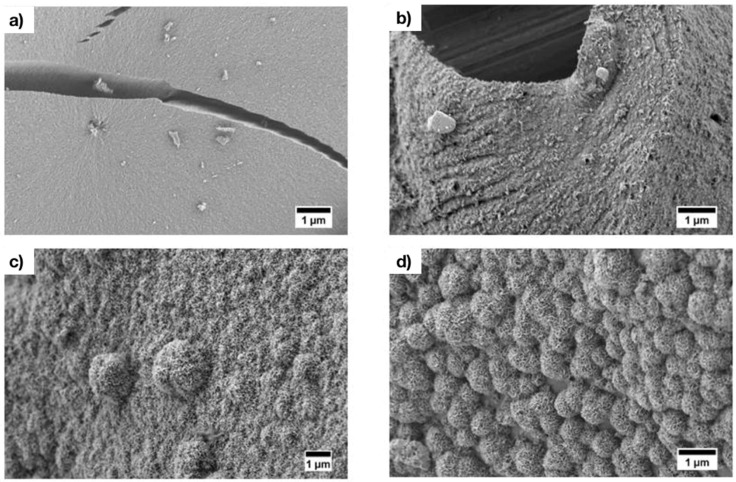
SEM micrographs of Ag-MBG after 0 days (**a**); 4 h (**b**); 1 day (**c**); and 3 days (**d**) of immersion in SBF. The typical cauliflower shape of hydroxycarbonate apatite (HCA) is already visible after 24 h of immersion.

After only 1 day of immersion in SBF, it was possible to observe the formation of a hydroxyapatite layer on the surface of the sample by SEM (Figure 3). After 3 days of immersion, the surface was homogeneously covered by a layer of hydroxycarbonate apatite (HCA).

**Figure 4:**
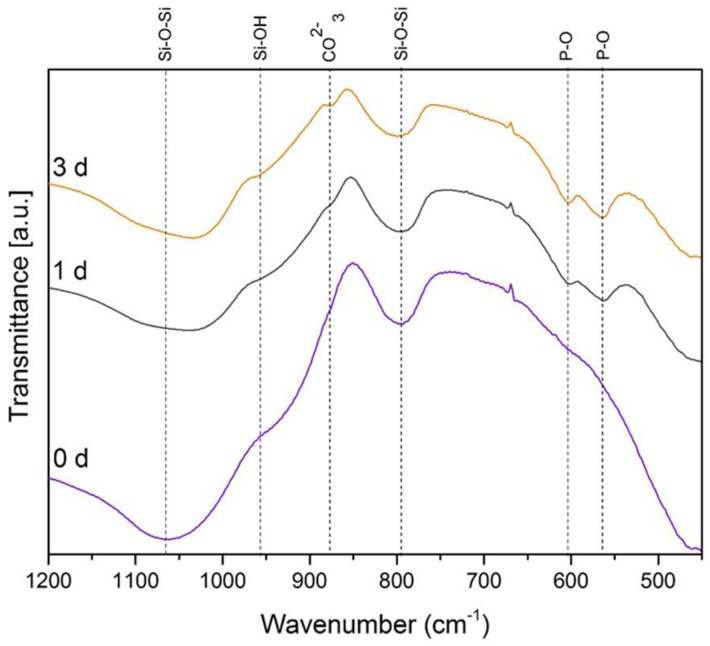
FTIR spectra of Ag-MBG before and after immersion in SBF at different time points; relevant bands are indicated in the spectra and discussed in the text.

The qualitative assessment of the presence of a HCA layer on the samples was further confirmed by FTIR, XRD, and Raman spectroscopy. Results after 7 days of immersion (not reported) did not show significant differences with respect to the ones reported after 3 days of soaking in SBF. Specifically, on the FTIR spectra (Figure 4), the P–O bending bands located at 560 and 604 cm^−1^ can be attributed to the phosphate group and are considered the main characteristic peaks of HCA. For this reason, they are used to confirm the formation of a HCA layer on the surface of the sample [30].

After immersion in SBF for 1 day, the two P–O bending peaks located at 560 and 604 cm^−1^ and a new carbonate band at 874 cm^−1^ are detected. Increasing the soaking time in SBF, the intensity of the peaks strengthens. This suggests not only the formation of an apatite-like layer but also that the layer is similar in terms of composition to the apatite phase in bones [30,31]. In comparison to the FTIR spectrum before immersion in SBF, a variation of three peaks centered at 800 cm^−1^, 960 cm^−1^, and 1070 cm^−1^ is noticed. Two of these peaks, namely the ones at 800 cm^−1^ and 1070 cm^−1^, correspond to the stretching of Si–O–Si, while the third one can be attributed to the stretching of silanol bonds (Si–OH) [30,31].

**Figure 5:**
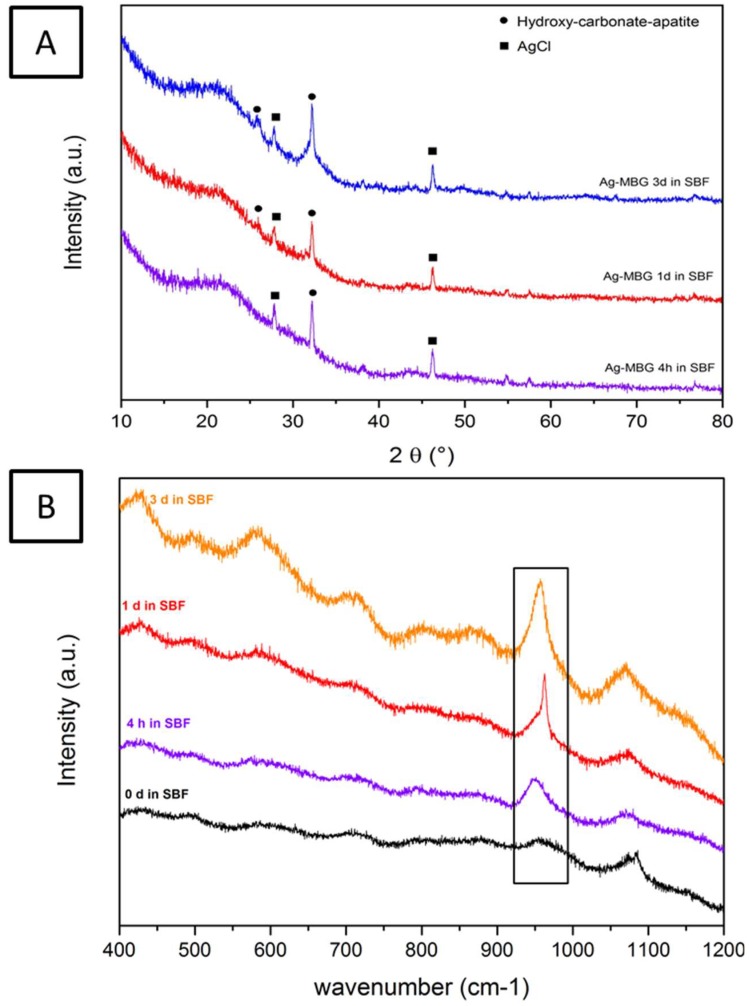
XRD (**A**) and Raman spectroscopy (**B**) results on Ag-MBG before and after immersion in SBF.

Furthermore, XRD and Raman spectroscopy analyses performed on samples previously immersed in SBF for up to 3 days confirmed the results obtained with FTIR. As previously discussed and as can be assessed in Figure 5A, XRD spectra of as-produced samples contain a very broad peak for 2θ values between 20° and 35°. However, after performing SBF test, in comparison with the XRD spectrum reported in Figure 1, it is possible to identify two new peaks at around 26° and 32°, which can be respectively assigned to the (002) and (112) reticular planes of hydroxyl carbonate apatite (HCA). Moreover, the peaks around 28° and 46° can be assigned to the (111) and (220) reticular planes of AgCl. It is likely that during the bioactivity test, Ag-MBG releases Ag^+^ ions which react with the chlorides in the SBF solution, thus forming an AgCl precipitate on the surface of the glass particles [13]. Raman spectra present a strong characteristic peak at 960 cm^−1^ that sharply increases over time and corresponds to the P–O symmetric vibration of the PO_4_^3−^ group (Figure 5B). The amount of HCA formed on the surface of the samples can be estimated, to a first approximation, by comparing the magnitude of the characteristic peak with the magnitude of the peaks in the spectrum of the 0-day sample. It can be observed that the 960 cm^−1^ peak increases with increasing immersion time in SBF, suggesting that the amount of formed HCA increases with time. The amount of HCA is also confirmed by the weaker peaks corresponding to asymmetric bending (438 and 454 cm^−1^) and asymmetric stretching (1078 cm^−1^) of the PO_4_^3−^ groups [13,32].

A qualitative assessment of the possible shielding of the Ag^+^ release caused by the formation of a thick hydroxycarbonate apatite on the samples was performed by EDX spectroscopic mapping on SBF preconditioned samples (3 days). The results are presented in the Figure S1, showing that after 3 days of immersion in SBF, silver was still detected, suggesting that the HCA layer does not shield the release of Ag^+^ from the MBG.

### 3.3. Ion Release

**Figure 6:**
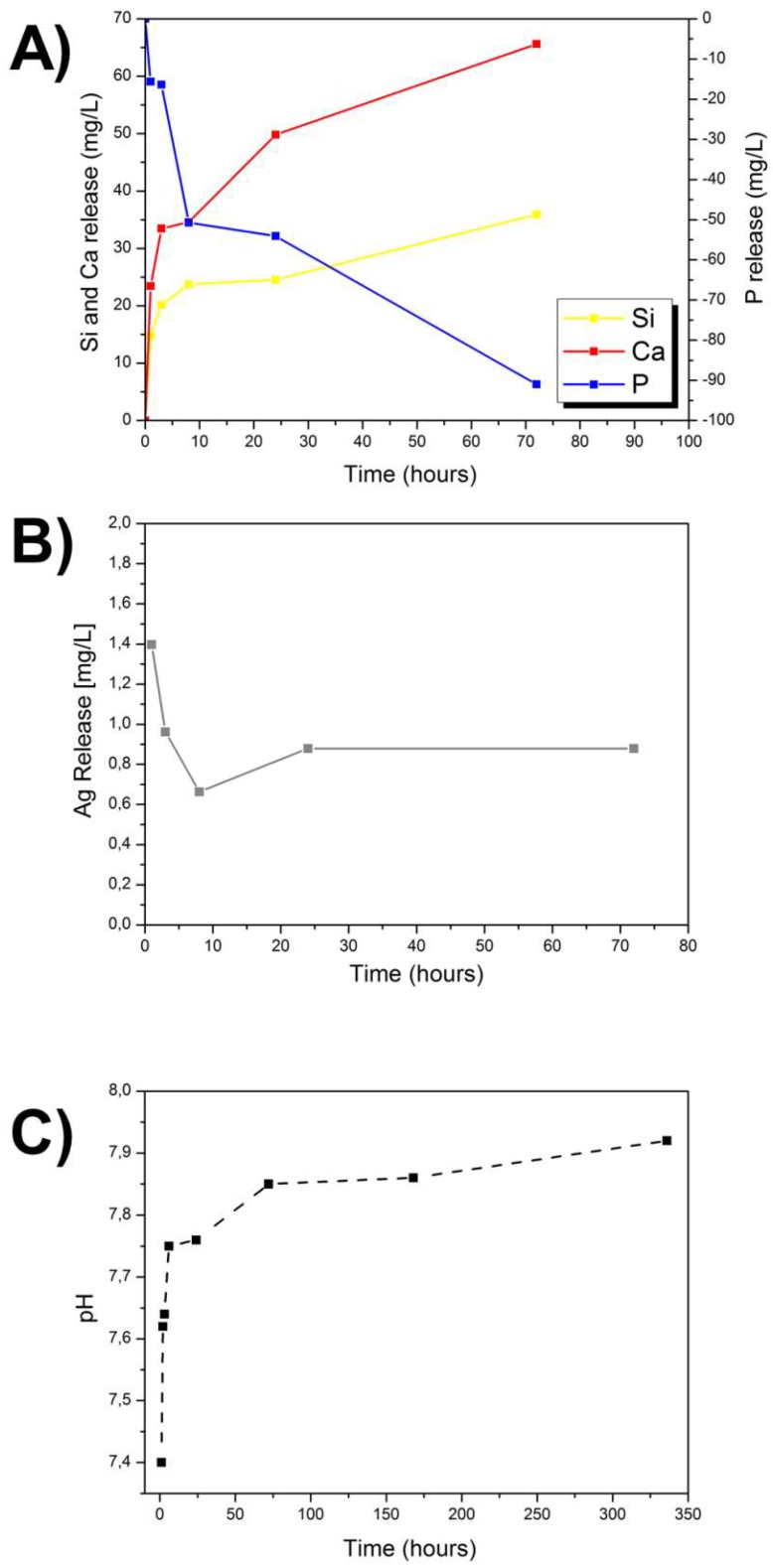
Profiles of ion release as function of time in SBF for Ag-MBG: Si, Ca, and P (**A**) and Ag (**B**) as a function of time. The pH variation of the solution is also shown (**C**).

The changes in Si, Ca, P, and Ag ion concentrations during the first 72 h of soaking in SBF are summarized in Figure 6. Glass dissolution took place in the first hours after immersion in SBF, as shown by the increase in pH and the continuous ion release [6]. After 3 days of immersion in SBF, around 36 mg L^−1^ of Si was released from the Ag-MBG. The Ca concentration after 8 h reached 140 mg L^−1^ and after 3 days it was found to be ~170 mg L^−1^. The concentration of phosphorus decreased over time due to the deposition of the CaP layer on the surface of the sample. The concentration of silver decreased in the first hours of immersion in SBF and after 3 days reached around 0.9 mg L^−1^. The decrease of the ion release might also be influenced by the formation of insoluble AgCl, which precipitates on the surface of the glass particles, as revealed by XRD analysis.

### 3.4. Antibacterial Studies

**Figure 7:**
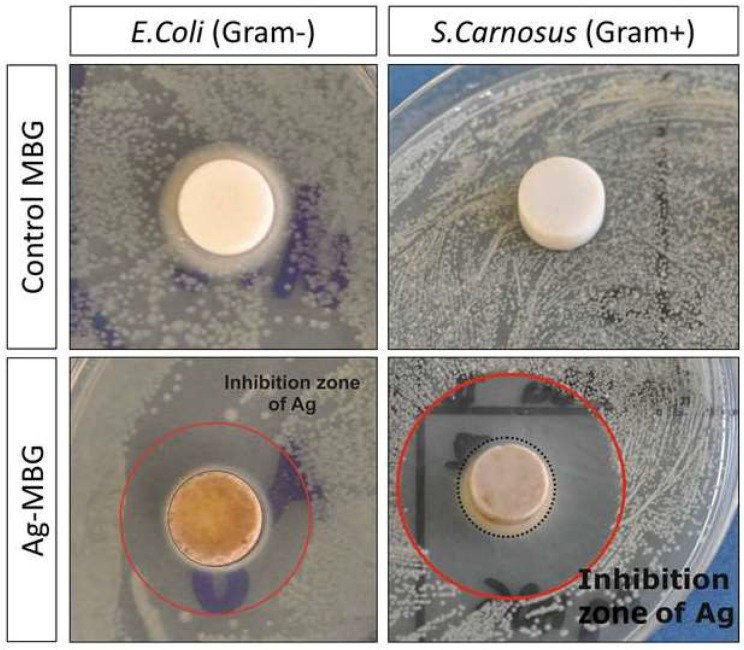
Results of antibacterial test showing the effect of Ag-MBG after 24 h of incubation against Gram-positive (*S. Carnosus*) and Gram-negative (*E. Coli*) bacteria.

Figure 7 shows antibacterial test results against *S. Carnosus*, Gram-positive, and *E. Coli*, Gram-negative, bacteria after 24 h of incubation. In both cases, an inhibition zone where bacteria are not present around the glass pellets is clearly visible. Using ImageJ, it was possible to calculate the diameter of the halo around the samples. In the case of *S. Carnosus*, it reached 1 cm; in the case of *E. Coli*, 0.5 cm. According to the Standard SNV 195920-1992, an inhibition zone greater than 1 mm is considered suitable for antibacterial activity [33,34]. Since the only difference between samples with and without antibacterial effects is the presence of silver, the results suggest that the antibacterial action is indeed induced by the progressive release of Ag^+^ from the MBG.

### 3.5. Incorporation of Ag-MBG Particles in Electrospun PCL fibres

**Figure 8:**
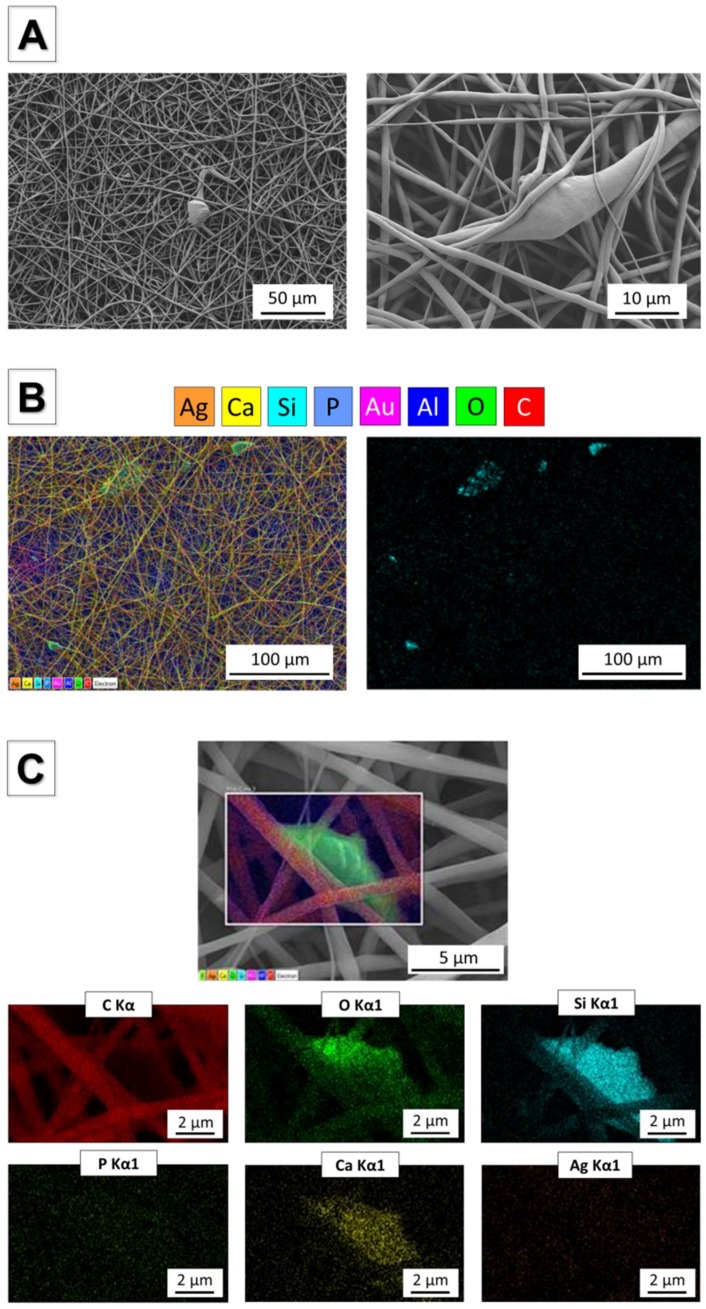
SEM micrographs showing incorporation of Ag-MBG particles in electrospun PCL fibres (**A**) and EDX analysis confirming the presence of Ag-MBG in the fibres (**B** and **C**).

Homogeneous bead-free fibrous mats were obtained starting from suspensions of PCL and Ag-MBG particles. SEM and EDX analyses are reported in Figure 8A–C. Ag-MBG particles were homogeneously dispersed in the PCL matrix, as shown in Figure 8A, in particular showing distribution of Si on the fibres. At higher magnification, it is possible to identify the Ag-MBG composition. The calculated average fibre diameter is 1.1 ± 0.2 μm, which is comparable with the value obtained from the neat PCL fibres [35], confirming that the presence of Ag-MBG particles did not affect the average fibre diameter.

### 3.6. 45S5 Bioactive Glass Scaffolds Coated by Ag-MBG

**Figure 9:**
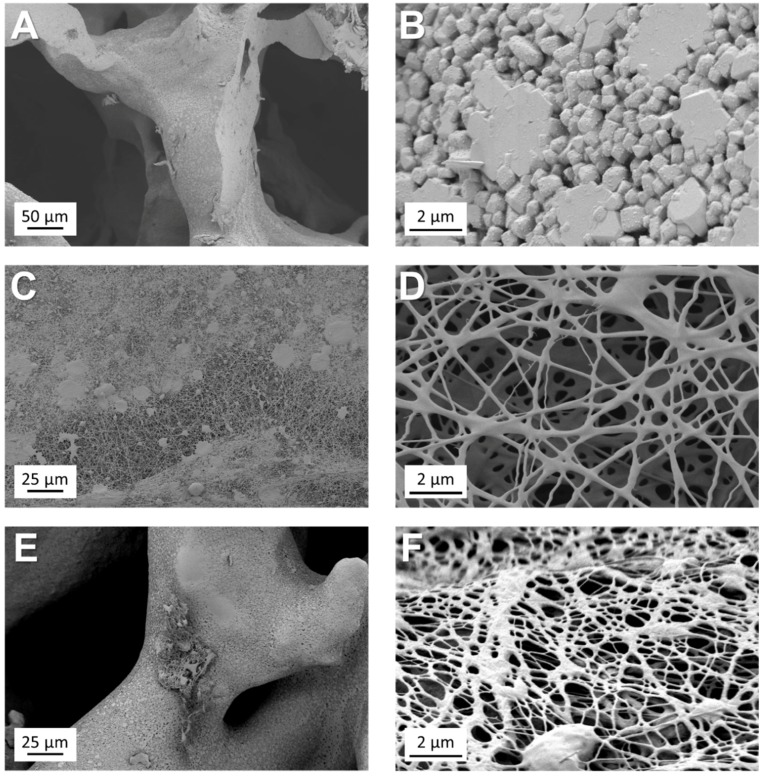
SEM micrographs of an uncoated foam replica scaffold (**A**,**B**) and of a Ag-MBG fibre-coated BG scaffold before (**C**,**D**) and after calcination (**E**,**F**).

The fully characterised mesoporous glass was used to coat bioactive glass-based scaffolds using the electrospinning technique. The obtained scaffolds were characterised via SEM. The results of the morphological analysis reported in Figure 9 show that foam replica scaffolds exhibit a well-interconnected macroporosity of several hundreds of microns, optimal for the infiltration and migration of cells and the subsequent growth of tissue. In addition, the electrospun coating treatment enriches the scaffold with smaller (submicrometric) features that can be used by cells as anchoring points, thus enhancing the ability of the scaffold to trigger cell attachment and adhesion. The calculated pore area of the electrospun top layer is around 0.15 μm^2^. Comparable data were obtained from both samples before and after calcination. The calcination step does not have critical consequences on the morphology of the fibres, even if the fibrous top layer does not homogeneously cover the BG scaffold surface. The result of the foam replica method followed by coating via electrospinning is a highly hierarchical scaffold structure that should be capable of stimulating and guiding the cell growth on many dimensional levels simultaneously, thus combining chemical and topographical stimuli [36].

## 4. Discussion

In the present work, a silver-containing mesoporous bioactive glass (Ag-MBG) was synthesised and characterised. We investigated its ability to develop a biologically active hydroxycarbonate apatite (HCA) surface layer, which mimics the mineral phase of bone [17,24] and its antibacterial properties both against Gram-positive (*S. Carnosus*) and Gram-negative (*E. Coli*) bacteria. Moreover, the elemental analysis performed on the Ag-MBG formulation confirmed that the glass preparation was optimally degradable and capable of releasing silver ions both with an initial burst release and a longer term sustained release. This type of release profile could be very beneficial for antimicrobial biomedical applications since it can strongly inhibit initial bacterial growth preventing later colonization of the device at the same time.

A feasibility study of possible applications of the synthesised Ag-MBGs was carried out with the production of Ag-MBG electrosprayed coating on bioactive glass-based scaffolds. The samples were produced by a foam replica technique [17] using natural marine sponges as a sacrificial template [20]. Marine sponges have been shown to be ideal substrates for the foam replica method, producing scaffolds with better balance between topography, porosity, and mechanical properties compared to scaffolds produced from polyurethane templates. Moreover, submicron composite mats (Ag-MBG/PCL) were also investigated.

As shown by the TEM micrograph (Figure 1B), an ordered MBG was obtained. The pore size was calculated to be around 7.5 nm, a value that is in the standard range of mesoporosity (2–50 nm). The mesopore size is a key feature in the design of a controlled drug delivery platform based on MBGs [9]. In fact, if the size of the molecule to be delivered is smaller than the pore diameter, it will penetrate inside the pores, otherwise it will only adsorb on the outer surface [9]. The nature of the adsorption will then affect the release behaviour. Therefore, it is important to characterise the mesoporosity of MBGs in order to assess if it will be suitable to encapsulate and release a given drug. Many MBG-based drug-delivery systems are reported in the literature, delivering molecules with a variety of steric hindrances: from sub-nanometric cisplatin (~0.5 nm) [37] and alendronate (0.83 nm) [38] to ibuprofen (1.01 nm) [39] all the way up to bovine serum albumin (BSA, 6 nm × 10 nm) [40]. The MBGs produced in this study have a similar size to the one characterised by Vallet-Regí et al. [40], which showed ideal porosity for the delivery of BSA and possibly other small proteins (e.g., growth factors). When the molecule to be loaded is far smaller than the pore diameter, most of the drug molecules will not be retained inside the pores because only some of them can directly interact with the pore walls. In this case, the main factor is the amount of retained molecules will be the specific surface area of the matrix.

The results of nitrogen physical analysis, carried out to determine the specific surface area of the MBG, showed that the BET surface area is 345 m^2^/g with a total pore volume of 0.423 cm^3^/g (calculated at P/P0 = 0.99). This result is relatively low compared to similar systems reported in literature [9,41]. Thus, when planning to combine Ag-MBG, for instance, with an antibiotic such as amoxicillin (1.1 nm) or gentamicin (0.9 nm), the main goal is to increase the surface area. When necessary, changes in pore size and surface area can be induced by changing the chain length of the surfactant, employing polymeric structure-directing agents or solubilizing auxiliary substances into micelles [9,40].

Acellular bioactivity tests in SBF were performed and after only 1 day of immersion it was possible to detect the formation of a bone-like hydroxyapatite layer on the surface of the sample. Further evidence supporting this finding was obtained by FTIR, XRD, and Raman spectroscopy. The rapid formation of HCA on the surface of the Ag-MBG anticipates that the material can rapidly bond to bone in vivo. This result is a significant improvement over previous studies using melt-derived, Ag-containing BG scaffolds, in which HCA formation was assessed only after 14 days in SBF. Also, it is important to note that our results confirmed, as already proposed by Bellantone et al. [15], that silver doping does not hinder the bioactivity of Ag-MBG, as it occurs for instance with zinc-doped BGs [42]. Not only is the ability of triggering biomineralization not altered in Ag-MBG compared to the undoped control, but also EDX spectroscopy qualitatively confirmed that the formation of hydroxycarbonate apatite does not shield the release of Ag^+^ from the MBG. Silver is also present on the samples after 3 days in SBF, suggesting that the formation of a HCA layer does not interfere with the Ag^+^ ion release. A similar result was obtained with ICP-OES, which confirmed that Ag-MBG is characterised by a steady and controlled release of silver in the first 3 days of immersion in SBF with a concentration of 0.9 mg L^−1^. Only small fluctuations occur and they are probably due to the formation of insoluble AgCl.

Preliminary antibacterial halo tests showed promising results against both Gram-positive (*S. Carnosus*) and Gram-negative (*E. Coli*) bacteria. After 24 h of incubation, both bacterial strains showed evidence of a significant inhibition zone where bacteria did not grow. The outcome of the bacterial culture is consistent with the amount of released silver, which showed values above the minimal inhibitory concentration (MIC) reported by previous studies on the topic [15,43]. However, as Sengstock et al. [43] reported, the toxic range of silver ions against prokaryotes is very close to the one against eukaryotes. In the near future, detailed studies will be carried out in order to assess and characterise the effect of Ag-MBG on mammalian cells and to determine the cytocompatibility levels.

Driven by the promising results in terms of bioactivity and antibacterial effect, the versatility of Ag-doped ordered mesoporous bioactive glass for the development of new technologies for bone and soft-tissue engineering was assessed. Homogeneous bead-free fibre mats were obtained by dispersing Ag-MBG particles in a solution of PCL dissolved in benign solvents for electrospinning (Figure 8). SEM confirmed the homogeneous embedding of Ag-MBG particles within PCL fibres. These preliminary results regarding the versatility of the synthesised Ag-MBG and the feasibility of producing composite electrospun fibres, by simply dispersing mesoporous particles in the polymeric solution before the electrospinning, are promising for pursuing the development of novel dual drug-delivery systems [35]. Additionally, results showed that Ag-MBG can be used as the main component of a nanostructured coating for bone-tissue-engineering porous scaffolds (Figure 9), enriching the bare scaffold with antibacterial properties and highly hierarchical topography suitable for the support of cell growth [44].

## 5. Conclusions

The use of Ag-doped ordered mesoporous glass was demonstrated as valid alternative for the development of antibacterial technologies for biomedical applications. The obtained MBG is characterised not only by a high specific surface area (345 m^2^/g), fast biomineralization behaviour, and a pore size of 7.5 nm but also by a satisfactory antibacterial effect against Gram-positive (*S. Carnosus*) and Gram-negative (*E. Coli*) bacteria. Moreover, the versatility of ordered mesoporous bioactive glasses was assessed by the production of homogeneous bead-free fibrous fibres and the coating of 45S5 bioactive glass scaffolds based on natural marine sponges. Further studies will be performed to characterise the cell-biology behaviour of the scaffolds in relevant conditions for assessing their potential for tissue-engineering applications.

## Figures and Tables

**Table 1:** Semiquantitative XRF analysis showing the chemical composition of Ag-MBG.

SiO_2_	CaO	P_2_O_5_	Ag_2_O
77.7	19.7	1.1	0.9
wt. %	wt. %	wt. %	wt. %

## Supplementary Materials

The following are available online at http://www.mdpi.com/1996-1944/11/5/692/s1, Figure S1: Images showing EDX mapping analysis on samples after 3 days of immersion in SBF.
