# Supplementary material for: Synthesis and Characterization of Silver-Doped Mesoporous Bioactive Glass and Its Applications in Conjunction with Electrospinning
Source: Materials (Basel). 2018 Apr 28;11(5):692. doi: 10.3390/ma11050692 (PMC5978069; doi:10.3390/ma11050692)
Supplement: Supplementary file 1 [file materials-11-00692-s001.pdf]

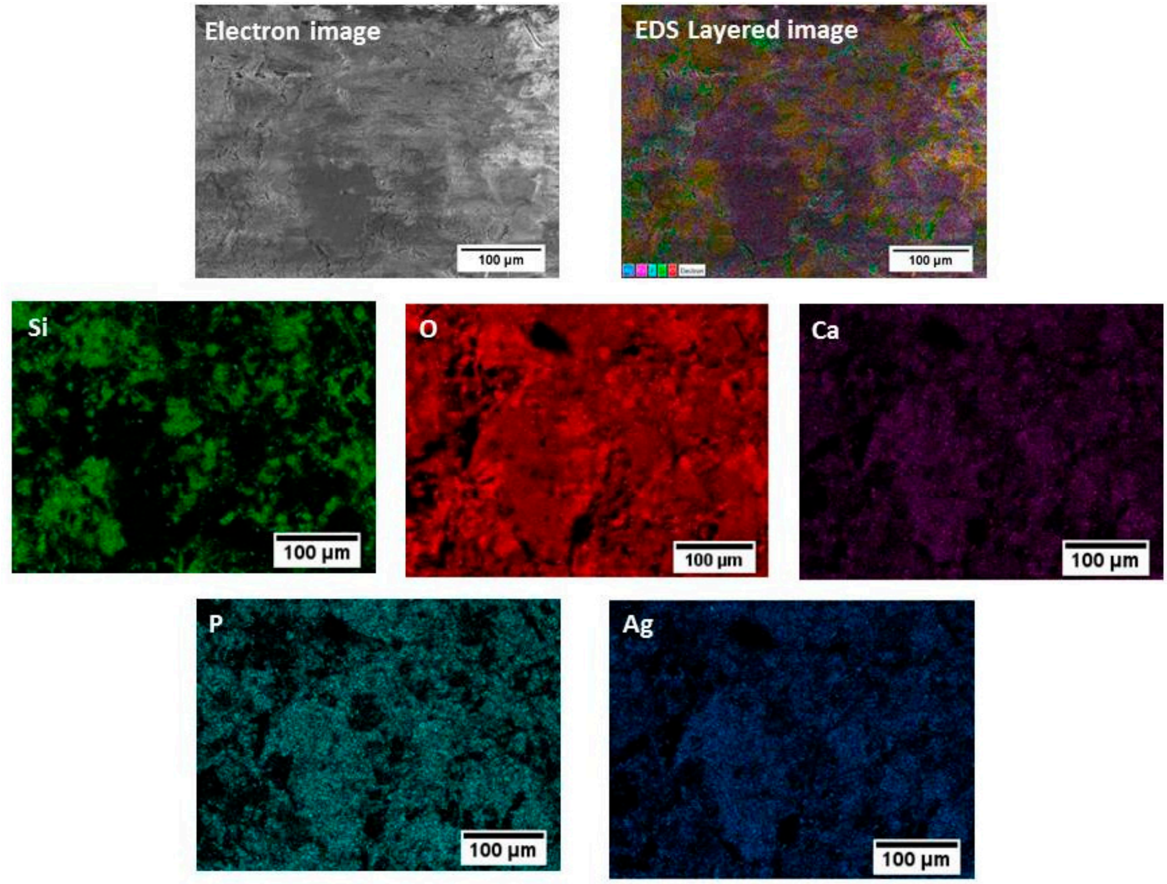

Supplementary information

Images showing EDX mapping analysis on samples after 3 days of immersion in SBF
